# Soluble factors from biofilms of wound pathogens modulate human bone marrow-derived stromal cell differentiation, migration, angiogenesis, and cytokine secretion

**DOI:** 10.1186/s12866-015-0412-x

**Published:** 2015-03-28

**Authors:** Catherine L Ward, Carlos J Sanchez Jr, Beth E Pollot, Desiree R Romano, Sharanda K Hardy, Sandra C Becerra, Christopher R Rathbone, Joseph C Wenke

**Affiliations:** Department of Extremity Trauma and Regenerative Medicine, United States Army Institute of Surgical Research, Fort Sam Houston, San Antonio, TX USA

**Keywords:** *Staphylococcus aureus*, *Pseudomonas aeruginosa*, Biofilm, Mesenchymal stromal cells, Wound healing, Differentiation, Angiogenesis

## Abstract

**Background:**

Chronic, non-healing wounds are often characterized by the persistence of bacteria within biofilms - aggregations of cells encased within a self-produced polysaccharide matrix. Biofilm bacteria exhibit unique characteristics from planktonic, or culture-grown, bacterial phenotype, including diminished responses to antimicrobial therapy and persistence against host immune responses. Mesenchymal stromal cells (MSCs) are host cells characterized by their multifunctional ability to undergo differentiation into multiple cell types and modulation of host-immune responses by secreting factors that promote wound healing. While these characteristics make MSCs an attractive therapeutic for wounds, these pro-healing activities may be differentially influenced in the context of an infection (i.e., biofilm related infections) within chronic wounds. Herein, we evaluated the effect of soluble factors derived from biofilms of clinical isolates of *Staphylococcus aureus* and *Pseudomonas aeruginosa* on the viability, differentiation, and paracrine activity of human MSCs to evaluate the influence of biofilms on MSC activity in vitro.

**Results:**

Exposure of MSCs to biofilm-conditioned medias of *S. aureus* and *P. aeruginosa* resulted in reductions in cell viability, in part due to activation of apoptosis. Similarly, exposure to soluble factors from biofilms was also observed to diminish the migration ability of cells and to hinder multi-lineage differentiation of MSCs. In contrast to these findings, exposure of MSCs to soluble factors from biofilms resulted in significant increases in the release of paracrine factors involved in inflammation and wound healing.

**Conclusions:**

Collectively, these findings demonstrate that factors produced by biofilms can negatively impact the intrinsic properties of MSCs, in particular limiting the migratory and differentiation capacity of MSCs. Consequently, these studies suggest use/application of stem-cell therapies in the context of infection may have a limited therapeutic effect.

**Electronic supplementary material:**

The online version of this article (doi:10.1186/s12866-015-0412-x) contains supplementary material, which is available to authorized users.

## Background

Effective wound healing is a highly coordinated, multistage process involving multiple host mechanisms, including inflammation, neovascularization, cell proliferation, extracellular matrix formation, and cellular maturation and remodeling. In contrast to acute wounds, which often heal with only minimal medical treatment, chronic wounds are primarily characterized by dysregulated inflammatory responses and cell defense mechanisms, which often result in delayed and/or non-healing of the wound [1]. The presence of microorganisms within tissues is a major risk factor for the development of chronic wounds. Recent studies evaluating the biodiversity within various types of chronic wounds demonstrated the presence of multiple species of bacteria, including *Staphylococcus aureus, Pseudomonas aeruginosa*, *Bacteroides spp., Peptostreptococcus, Enterococcus spp* [2,3]*.* In contrast to surviving in a predominately planktonic form, such as in vitro cultures, bacteria adapt to and persist within chronic wounds as a heterogeneous population that is predominately attached to host tissues, known as a biofilm [4,5]. To date numerous studies have demonstrated the presence of bacterial biofilms in various settings involving chronic human infections, indicating a causal relationship between the presence of bacterial biofilms and the development of these types of infection. For example, in a sample of patients with chronic, non-healing cutaneous wounds, over half were characterized as containing biofilm [4]. Similarly, recent reports evaluating nonunion of long bone fractures have also shown that the presence of biofilms was frequently associated with non-osseous union [6]. Although the precise mechanisms through which biofilm bacteria contribute to and promote wound chronicity is not completely understood, a few studies have demonstrated that soluble factors released from the biofilm can negatively impact cell function [4,5,7]. Studies evaluating interactions between biofilm communities and cells involved in wound healing will be central to further our understanding of how these processes may be perturbed during infection.

Regenerative cells known as mesenchymal stem cells, multipotent mesenchymal stromal cells, and marrow stromal cells (hereafter referred to as “MSCs” [8]) contribute to wound healing directly through differentiation [9,10], and indirectly through the release of paracrine factors, such as stromal derived factor 1 (SDF-1) [11,12] and vascular endothelial growth factor (VEGF) [13]. Additionally, MSCs have also been shown to have antimicrobial properties through activities mediated by the release of the antimicrobial peptide LL-37 [14-16]. The combined properties of MSCs make them a critical element of intrinsic wound healing, and a highly attractive candidate for cell based therapies for tissue repair and regeneration. To date, numerous studies have evaluated the potential therapeutic applications of MSCs for tissue regeneration. However, the vast majority of these studies have primarily utilized wound models that lack an infectious component. This is a particularly important lapse, as the functional activity of MSCs may be altered in the presence of inflammatory mediators produced by the host as well as bacterial components that may accumulate within a chronic wound. In support of this, recent studies have demonstrated differential effects to normal healing and differentiation of MSCs in the presence of purified microbial cell wall components (e.g., Gram-negative lipopolysaccharide (LPS) and Gram-positive lipoteichoic acid, (LTA)) [17,18], inflammatory cytokines [19], peripheral blood mononucleated cells (PBMCs) [20], and heat-inactivated bacteria [21]. Consequently, these studies indicate that the ability of MSCs to promote healing can be negatively affected in the context of the wound environment.

Given the importance of bacterial biofilms in the development of chronic wounds, the ability of these communities to inhibit wound healing, and the likely interactions with host cells such as MSCs, herein we evaluated the effect of soluble factors from biofilms of clinical isolates of *S. aureus* and *P. aeruginosa* on the viability, differentiation, and paracrine activity of human bone marrow stromal cells (hBMSCs) in vitro.

## Methods

### hBMSCs and cell culture

Primary human bone marrow stromal cells (hBMSCs, STEMCELL Technologies, Vancouver, Canada) were maintained in Dulbecco’s modified Eagle’s medium (DMEM; Gibco®, Life Technologies, Grand Island, NY) supplemented with 10% fetal bovine serum (FBS) and 10 U penicillin mL^−1^ and streptomycin 10 μg mL^−1^ at 37°C in 5 % CO_2_. For all studies described herein, hBMSCs between passages 1–4 were used.

### Bacterial strains and growth conditions

UAMS-1 (ATCC strain 49230) is a methicillin-susceptible *S. aureus* clinical strain of the USA 200 clonal group and a well characterized osteomyelitis isolate [22]. *P. aeruginosa*, strain SAMMC-418, is a multidrug-resistant, pulsed-field type 2, clinical wound isolate selected from a strain collection at the San Antonio Military Medical Center (SAMMC, Ft. Sam Houston, TX) [23]. Both strains used in this study have been previously characterized for biofilm formation. Bacteria were cultured in Cation-Adjusted Mueller Hinton Broth (MHB-II) or on blood agar plates at 37°C.

### Biofilm formation and preparation of biofilm-conditioned media (BCM)

Biofilm formation and generation of conditioned media were performed as previously described [24,25]. Briefly, 500 μL of a 1:100 dilution of overnight bacterial cultures (~10^6^ CFU/mL) were added to transwell tissue culture inserts (0.4 μm pore size, Corning Inc, Corning, NY) and grown under static conditions for 48 hr at 37°C. Culture inserts were washed, placed into DMEM, and maintained at 37°C for up to 4 days. BCM was collected daily and replaced with fresh media. Consistent with previous studies and to reduce variations between individual collections of BCM, individual fractions were pooled [24,25]. Pooled BCMs were filter-sterilized using a 0.2 μm syringe filter (Fisher, Pittsburgh, PA) to remove any bacteria, pH adjusted to 7.4, and stored at −80°C. Sterility of collected BCM was assessed by spot plating 10 μL aliquots of BCM onto MHB-II plates and incubating the plates overnight at 37°C.

### Enumeration of bacteria and protein within biofilms

To evaluate the relationship between bacteria within biofilms and level of protein present within the conditioned medias over time, biofilms of *S. aureus* UAMS-1 and *P. aeruginosa* SAMMC-418 were grown in transwells as described above and viable bacteria along with total protein within the collected medias were measured daily for up to five days. Bacteria were enumerated by removing the polycarbonate membrane of the transwell, followed by sonication of the entire membrane in 1 mL of PBS and enumeration by plating serial dilutions on blood agar plates. Protein corresponding to the same time point and within the media was quantified using a bicinchonic acid (BCA) assay (Pierce, Rockford, IL) following the manufacturer’s protocol.

### Preparation of planktonic-conditioned media (PCM)

Planktonic bacterial cultures of *S. aureus* and *P. aeruginosa* were grown under conditions to produce similar cell densities of the biofilm cultures. Briefly, individual bacterial colonies from overnight plate cultures were inoculated into MHB-II and grown to early stationary phase (~10^9^ CFU/mL). Following growth in liquid culture, bacteria were harvested by centrifugation and resuspended in sterile DMEM, then incubated at 37°C with agitation for an additional 24 hr. Following overnight incubation, planktonic bacteria were then harvested by centrifugation and the supernatants were collected, filter sterilized and adjusted to pH 7.4 prior to use in experimental assays. Similar to the BCM, the PCM was spot plated onto MHB-II plates and incubated overnight at 37°C to ensure sterility of supernatants.

### Cell viability

Cells were seeded into 48-well plates (at 5 × 10^3^ cells/cm^2^), grown to ~50% confluence, and exposed to either purified bacterial cell wall components, including lipoteichoic acid (LTA) from *Staphylococcus aureus* or lipopolysaccharide (LPS) from *Eschrichia coli* 0111:B4 (Sigma Aldrich, St. Louis, MO) at 10 μg/mL [26], PCM or BCM of *S. aureus* and *P. aeruginosa* for up to 7 days in vitro. As a control for the treatment groups, cell media supplemented with PBS at similar concentrations were used for comparison (vehicle control). Viability was assessed for up to 7 days post-exposure using a LIVE/DEAD^©^ assay kit (Life Technologies™, Grand Island, NY) by measuring fluorescence at EX_485nm_/EM_530nm_ using a microplate reader (SPECTRAmax M2, Molecular Devices, Sunnyvale, CA) per the manufacturer’s instructions. Cell viability was expressed as a ratio of an untreated, media only, growth control for each respective timepoint.

### Measurement of Caspase-3/7 activity

Caspase-3/7 activity in hBMSCs following exposure to BCM was determined using the SensoLyte™ Homogenous AFC Caspase-3/7 Assay Kit (AnaSpec, San Jose, CA) for up to 7 days, as previously described [27]. Briefly, 25 μL of cell media from BCM-exposed hBMSCs was combined with the caspase-3/7 substrate solution to initiate an enzymatic reaction to cleave and generate a fluorophore. Fluorescence was detected at EX_380nm_/EM_500nm_ using a microplate reader. Caspase activity, measured by released caspase-3/7 found in the media, was expressed as a ratio of an untreated growth control. As in viability experiments, caspase activity was also evaluated in cells exposed to vehicle control, purified cell wall components, and PCM of both *S. aureus* and *P. aeruginosa*.

### Western blot analysis

Protein analysis was performed on cell lysates of hBMSCs exposed to BCM after 7 days in culture. Briefly, whole cell lysates were prepared by lysing cells with CelLytic™ M (Sigma-Aldrich, St. Louis, MO) followed by treatment with protease inhibitor cocktail (Sigma-Aldrich). Triplicate samples were pooled and concentrated using 3000 NMWL centrifugal devices (Millipore, Billerica, MA). Total cellular protein was determined by bicinchoninic acid (BCA) assay (Pierce). Whole cells lysates (20 μg) were separated on a 3-8% Tris-Acetate gel (NuPAGE®, Life Technologies, Grand Island, NY) and electrophoretically transferred to nitrocellulose membranes. Membranes were blocked with PBS containing 5% bovine serum albumin (BSA) and 0.1% Tween-20 for 1 hr and incubated overnight at 4°C with primary antibodies including; rabbit anti-human caspase-3, rabbit anti-human adiponectin, rabbit anti-human PPARγ, rabbit anti-human RUNX2, (1:1000; Cell Signaling Technology®, Danvers, MA), goat anti-human leptin, rat anti-human alkaline phosphatase (ALP) (0.1 μg/mL, R&D Systems, Minneapolis, MN), rabbit anti-human osteocalcin (OCN) (1:1000; abcam®, Cambridge, MA) and mouse anti-human Glyceraldehyde-3-phosphate dehydrogenase (GAPDH) antibody (1:500; Millipore, Billerica, MA). Following incubation, membranes were washed then incubated with the appropriate HRP-conjugated secondary antibody (1:3000–1:5000) for 1 hr at room temperature for detection of the proteins by chemiluminesence. Relative protein levels were determined by comparative densitometric analysis of western blot bands using the Odyssey Fc system (LI-COR Biosciences, Lincoln, NE). For each protein examined, the membrane initially probed was stripped, and the amount of actin was determined using antibodies against GAPDH. Relative levels of protein were determined by dividing the intensity of the tested protein band to that of GAPDH within the same lane and membrane.

### Adipogenic differentiation

Cells were cultured at 5 × 10^3^ cells/cm^2^ in 24-well plates and grown to ~80% confluence. For adipogenic differentiation, cells were cultured in growth media supplemented with isobutyl-methyl xanthine (50 mM), indomethacin (200 μM), insulin (1 μM), and dexamethasone (0.1 μM) (Sigma-Aldrich, St. Louis, MO). Differentiation control groups contained an equivalent volume of unsupplemented DMEM (as a vehicle control) as BCM in the experimental groups. Cells were cultured for up to 21 days, with fresh media changes every 3–4 days. At days 7, 14 and 21, cells were fixed in 10% formalin, stained for 10–15 min at room temperature with a working solution of Oil Red O stain (0.0035 g/mL in isopropanol, diluted 6:4 in deionized water), rinsed 3x with distilled water, and oil droplets were visualized by light microscopy at 40x magnification (Olympus IX71 microscope, Center Valley, PA)_._ For quantification of staining, Oil Red was extracted from cells using 100% isopropanol and absorbance at 500 nm was measured. Results were expressed as a ratio of an undifferentiated growth control. Protein expression levels of genes involved adipogenic differentiation, including adiponectin, PPARγ, and leptin, was also performed on hBMSCs after 7 days of differentiation by western blot analysis as described above.

### Osteogenic differentiation

For osteogenic differentiation, cells were cultured as above in growth media supplemented with ascorbate-2 phosphate (50 μM), β-glycerolphosphate (10 mM), and dexamethasone (0.1 μM) (Sigma-Aldrich, St. Louis, MO). At days 7, 14 and 21, cells were fixed with 10% formalin, stained with 2% w/v Alizarin Red S in deionized water (pH 7.0) for 20 min, washed with PBS, and calcium deposits were visualized by light microscopy at 40x magnification (Olympus IX71 microscope, Center Valley, PA). For quantification of staining, Alizarin Red was extracted from cells using 0.5 N HCl in 5% SDS solution and absorbance at 415 nm was measured. Results were expressed as a ratio of an undifferentiated growth control. Protein expression levels of genes involved osteogenic differentiation, including RUNX2, OCN, and ALP, was also performed on hBMSCs after 7 days of differentiation by western blot analysis as described above.

### RNA extraction and quantitative real-time PCR (qRT-PCR)

RNA extraction and isolation from hBMSCs exposed to BCM was performed as previously described [24]. First strand synthesis of cDNA was achieved with SuperScript III first-strand synthesis supermix with oligo-dT primers (Invitrogen^TM^, Grand Island, NY) for each RNA sample using a PTC-100 Thermal Cycler (GMI Inc, Ramsey, MN). For genes of interest, quantitative real-time polymerase chain reaction (qRT-PCR) was performed using a Bio-Rad C1000 system and analyzed using iQ5 software (BioRad, Hercules, CA) using 10 ng of RNA per reaction. Primers utilized in this study for genes of interest, including peroxisome proliferator-activated receptor gamma (*PPAR*γ), *adiponectin*, *leptin*, alkaline phosphatase (*ALP*), runt-related transcription factor 2 (*RUNX2*), and bone gamma-carboxyglutamic acid-containing protein (osteocalcin, *OCN*), were purchased from SABiosciences (Valencia, CA) and the amplification reactions were performed using SYBR Green Super Mix (BioRad, Hercules, CA) following the optimized conditions recommended by the manufacturer. Three independent biological experiments with three technical replicates were performed for each reaction. Transcript levels were normalized to GAPDH mRNA. Relative expression levels were calculated using the 2^–ΔCt^ method [28]. Relative fold differences in gene expression were calculated as a ratio of the normalized values compared to relative gene expression in undifferentiated controls.

### Capillary tube formation assay

Angiogenesis, as determined by capillary tube formation of hBMSCs, was assessed using a matrigel-based assay as previously described [29]. Briefly, individual wells of a 48-well tissue culture plate were coated with 100 μL of Matrigel (100 mg/mL, BD Biosciences, San Jose, CA) and allowed to polymerize for 1 hr at 37°C. Cells were seeded on top of the gels at 2 x 10^4^ cells/well in 200 μL of media containing BCM. At 2, 6, and 16 hours post-exposure, six images per well were taken at 40× and 100× magnification with an inverted microscope (Olympus IX71 microscope, Center Valley, PA) to analyze the development of capillary-like networks using phase contrast microscopy. Image analysis was performed using Image J V1.44p software (U. S. National Institutes of Health, Bethesda, MD). Capillary tube formation was assessed at each time point by counting the number of capillary-like tubules and by measuring tube length (μm) between connectors.

### Cell migration

Functional migration analysis of hBMSCs was performed using an Oris™ Cell Migration Assay-FLEX (Platypus Technologies, Madison, WI) according to the manufacturer’s instructions. Briefly, cells were seeded into black 96-well clear bottom microplates (5 × 10^3^cells/cm^2^) containing a rubber stopper at the plate center, creating a void circle (2 mm diameter) area. hBMSCs were grown to ~80% confluence at which time the stoppers and media were removed, and replenished with media containing BCM. Positive control wells containing growth media (0% BCM, with an equivalent volume of unsupplemented DMEM as the BCM experimental groups to represent a vehicle control) and negative control wells with stopper in place (depicting no migration) were maintained in the well plate. Cells were allowed to migrate for 3 days. Media was then removed and cells were stained with Vybrant® CFDA SE Cell Tracer dye (Invitrogen^TM^, Grand Island, NY). Wells were viewed at 40x magnification using an inverted microscope (Olympus IX71 microscope, Center Valley, PA) with a FITC filter to image the entire area of migration. Using Adobe® Photoshop® CS3 (San Jose, CA), images were converted to black and white, and threshold was recorded. Quantification was performed by calculating a ratio based on the threshold of the experimental well images to that of a full growth control (i.e., 100% confluency). This ratio was deemed as the amount of migration that occurred into the void area over the course of 3 days.

### Quantification of cytokines

Enzyme-linked immunosorbant assays (ELISAs) were performed to quantify cytokines in culture supernatants (media) from hBMSCs exposed to BCM for 1 and 3 days. ELISA kits for Tumor necrosis factor α (TNF-α) (R&D Systems, Minneapolis, MN), Interleukin-6 (IL-6) (R&D Systems, Minneapolis, MN), stromal cell derived factor 1a (SDF-1) (TSZ ELISA^TM^, Framingham, MA), vascular endothelial growth factor (VEGF) (Quantikine®, Minneapolis, MN), and human cathelicidin LL-37 (Hycult® Biotech, Netherlands) were performed according to the manufacturer’s instructions. Briefly, supernatants (media) were collected from individual wells containing hBMSCs exposed to BCM at days 1 and 3 (0.5 mL volumes), treated with protease inhibitor cocktail (Sigma-Aldrich, St. Louis MO), and stored at −80°C until analysis. Total protein in samples was quantified using a BCA assay (Pierce). Cytokine levels in supernatants were quantified by extrapolation from standard curves generated using recombinant protein for each respective cytokine, and determined concentrations were normalized to total protein content in each supernatant sample as determined by the BCA assay.

### Statistical analysis

Statistical analysis was performed using one-way ANOVA with a Tukey’s multiple comparison post hoc test for comparisons between groups. *P* values of <0.05 were considered to be statistically significant. All assays were performed in triplicate.

## Results

### Soluble factors from biofilms reduce the viability of hBMSCs, in part due to the activation of apoptosis

**Figure 1:**
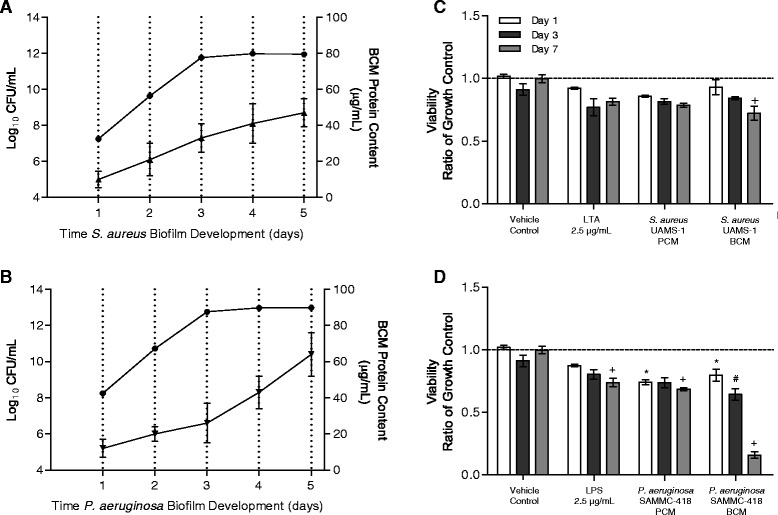
**Soluble factors from biofilms reduce viability in hBMSCs.** Enumeration of viable bacteria within biofilms **(A)** and measurement of released protein from biofilms **(B)** of *S. aureus* UAMS-1 and *P. aeruginosa* SAMMC-418 grown for up to 5 days. **(C)** Cell viability of hBMSCs over a 7 day period following exposure to media containing 25% PBS (vehicle control), lipoteichoic acid (LTA, 10 μg/mL at 25%, Gram-positive cell wall component), *S. aureus* UAMS-1 planktonic-conditioned media (PCM), and *S. aureus* UAMS-1 biofilm-conditioned media (BCM). **(D)** Cell viability of hBMSCs over a 7 day period following exposure to media containing 25% PBS (vehicle control), lipopolysaccharides (LPS, 10 μg/mL at 25%, Gram-negative cell wall component), *P. aeruginosa* SAMMC-418 PCM, and *P. aeruginosa* SAMMC-418 BCM. Values are expressed as a ratio of the untreated growth control (untreated hBMSCs exposed to complete growth media, indicated by dashed lines) and represent the average ± SEM. *,#,+ *p* < 0.05 compared to day 1, 3 and 7 controls, respectively.

Initial screening of the effect of soluble factors of biofilms on cell viability, was performed by exposing hBMSCs to biofilm factors of *S. aureus* and *P. aeruginosa* at increasing concentrations (0, 5, 10, 25, 50, 75, and 100%) in cell growth media for 24 hours. Based on these preliminary results, a concentration of 25% BCM was determined to provide optimal conditions to test the effect of BCM on hBMSC function without substantial loss of viability (i.e., to maintain a testable population for extended periods of time), and therefore this concentration was used throughout all future studies (Additional file 1: Figure S1). Exposure of hBMSCs to biofilm factors of *S. aureus* and *P. aeruginosa* at 25% was observed to have differential effects on cell viability during the 7 day exposure, as compared to the untreated growth control group (Figure 1C-D). Of note, no significant loss of cell viability was observed in cells exposed to media supplemented with PBS at 25% (vehicle control group) for up to 7 days, indicating that the observed effect was not the direct result of nutrient depletion but specific for the factors within the conditioned medias. Biofilm-derived factors from *P. aeruginosa* were observed to have an overall greater negative effect on cell viability compared to factors derived from biofilms of *S. aureus*, with significant loses in cell viability observed at 1, 3, and 7 days post-exposure compared to the untreated growth controls. *S. aureus* BCM was only observed to have a significant negative effect on cell viability after 7 days. These observed differences of the effect of BCMs on cell viability may have been in part due to the differences in bacterial number observed within biofilms as well differences in total protein content with the respective BCMs (Figure 1A-B). In contrast to the effects of BCM on hBMSCs, exposure to purified bacterial cell wall components, including LTA and LPS, or the respective PCM of *S. aureus* and *P. aeruginosa* were observed to only have a moderate effect on cell viability (Figure 1C-D).

**Figure 2:**
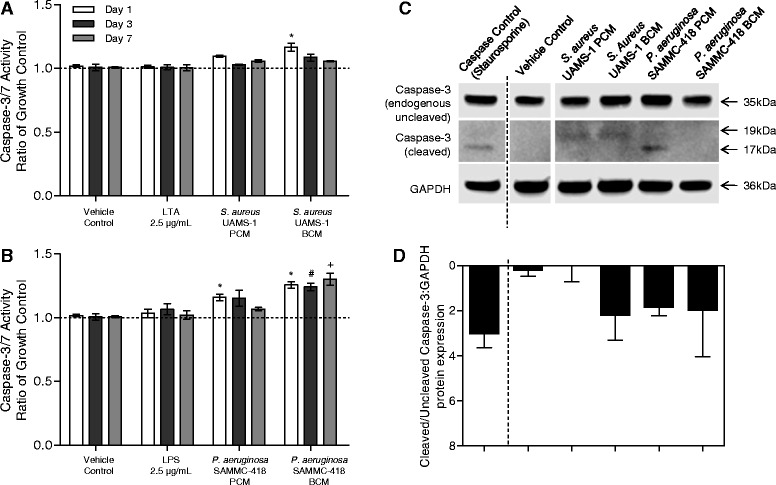
**Soluble factors from biofilms increase caspase activity in hBMSCs. (A)** Caspase-3/7 activity of hBMSCs over a 7 day period following exposure to media containing 25% PBS (vehicle control), lipoteichoic acid (LTA, 10 μg/mL at 25%, Gram-positive cell wall component), *S. aureus* UAMS-1 planktonic-conditioned media (PCM), and *S. aureus* UAMS-1 biofilm-conditioned media (BCM). **(B)** Caspase-3/7 activity of hBMSCs over a 7 day period following exposure to media containing 25% PBS (vehicle control), lipopolysaccharides (LPS, 10 μg/mL at 25%, Gram-negative cell wall component), *P. aeruginosa* SAMMC-418 PCM, and *P. aeruginosa* SAMMC-418 BCM. Values are expressed as a ratio of the untreated growth control (untreated hBMSCs exposed to complete growth media, indicated by dashed lines) and represent the average ± SEM. *,#,+ *p* < 0.05 compared to day 1, 3 and 7 controls, respectively. **(C)** Western blot analysis of Caspase-3 antibody on hBMSC lysates, illustrating the presence of the endogenous uncleaved form (35 kDa) and the activated cleaved form (17-19 kDa). **(D)** Semiquantitative analysis of the ratio of activated (cleaved) to inactive (uncleaved) levels of caspase-3 as determined by densitometric analysis. Protein levels were normalized to levels GAPDH probed on the same membrane. Staurosporine, an apoptic inducing agent was included as a positive control for apoptosis.

Consistent with the results from the viability assays, cellular caspase 3/7 activity was observed to increase following exposure to BCM of both *S. aureus* and *P. aeruginosa* at day 1 post exposure, and for *P. aeruginosa* at days 1, 3 and 7, in part explaining the reduced cell numbers observed over time (Figure 2A-B). In support of this, western blot analysis of activated caspase activity in pooled cell lysates demonstrated elevated levels of caspase in BCM-exposed groups (Figure 2C-D). As observed in previous experiments, exposure of cells to LTA, LPS, and the respective PCMs of both strains were observed to have little to moderate effect on cell apoptosis compared to the BCM. Collectively these observations support the notion that biofilms release factors that are not observed in the planktonic form, justifying further analysis of the BCM on the activity of hBMSCs.

### Multilineage differentiation potential of hBMSCs is decreased in the presence of soluble factors from biofilms

**Figure 3:**
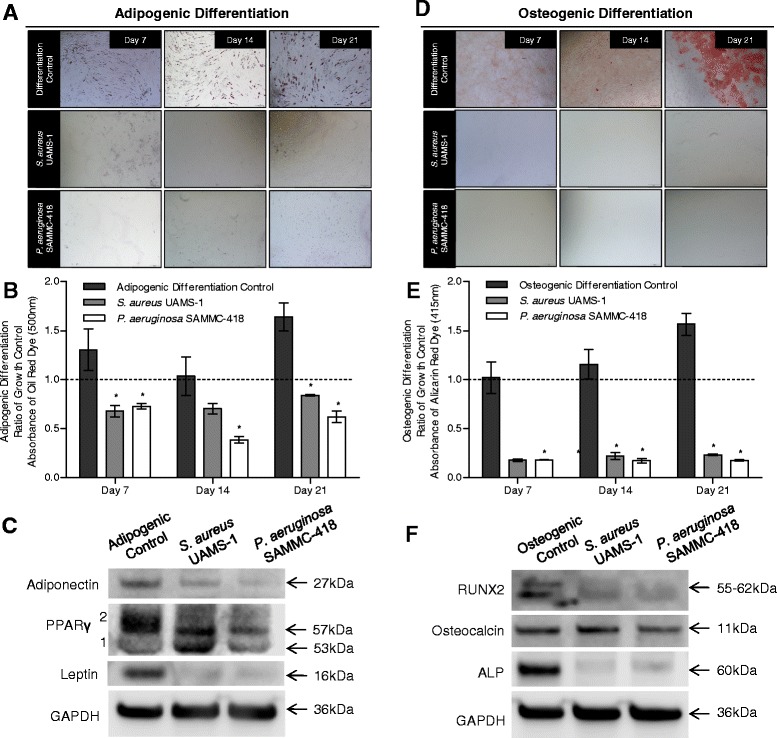
**Soluble factors from biofilms differentially affect adipogenic and osteogenic differentiation of hBMSCs in vitro. A)** Light microscopy images of hBMSCs stained with Oil Red O (red, 40x magnification) after adipogenic differentiation on days 7, 14 and 21 in the presence of BCM (25%) from *S. aureus* or *P. aeruginosa.* **B)** Quantification of Oil Red O stain (OD_500nm_) to detect intracellular oil droplets following adipogenic differentiation determined by extraction and solubilization of the dye. **C)** Western blot analysis of adipogenic proteins: adiponectin, peroxisome proliferator-activated receptor gamma (PPARγ), and leptin. **D)** Light microscopy images of hBMSCs stained with Alizarin Red S (red, 40x magnification) after osteogenic differentiation on days 7, 14 and 21 in the presence of BCM (25%) from *S. aureus* or *P. aeruginosa.* **E)** Quantification of Alizarin Red S stain (OD_415nm_) to detect intracellular calcium deposits following osteogenic differentiation. **F)** Western blot analysis of osteogenic proteins: Runt-related transcription factor 2 (RUNX2), bone gamma-carboxyglutamic acid-containing protein (osteocalcin), and alkaline phosphatase. Quantification results are expressed as a ratio of non-differentiated growth controls (dashed line) at each timepoint; *p < 0.05 compared to differentiation control at each respective timepoint.

A central feature of multipotent mesenchymal stromal cells is their ability to undergo multilineage differentiation and contribute to regeneration of injured tissues. In the presence of BCM from *S. aureus* and *P. aeruginosa*, the multilineage differentiation potential of hBMSCs was significantly reduced. At 7, 14 and 21 days post-differentiation, cells exposed to BCM from either *S. aureus* or *P. aeruginosa* were significantly impaired in their ability to differentiate into mature adiopocytes and osteocytes compared to the unexposed differentiation controls (Figure 3). Visual examination of cells, as demonstrated in representative images, for presence of oil droplets and intracellular calcium deposits, markers of adipogenic and osteogenic differentiation, respectively, were reduced compared to the differentiation control groups (Figure 3A and D). Measurement of the ratio of solubilized oil red and alizarin red to undifferentiated growth controls also demonstrated decreases in cell differentiation (Figure 3B andE). Consistent with this visual decrease in differentiation capability, independent gene (Tables 1 and 2) and protein expression analysis (Figure 3C and F, Additional file 2: Figure S2) of adipogenic and osteogenic markers also demonstrated a reduced differentiation capacity of hBMSCs following exposure to BCM.

**Table 1:** **Relative expression of genes involved in adipogenic differentiation**

	**Day 3**		**Day 7**	
	**Relative expression**	**Fold difference**	**Relative expression**	**Fold difference**
***PPAR*** **γ**				
**Differentiation control**	0.70 ± 0.37	*14.45*	0.11 ± 0.71	*4.72*
***S. aureus*** **UAMS-1**	0.29 ± 0.26	*6.02*	0.19 ± 0.50	*8.19*
***P. aeruginosa*** **SAMMC-418**	0.50 ± 0.29	*10.31*	0.20 ± 0.74	*8.53*
***Adiponectin***				
**Differentiation control**	0.00 ± 1.42	*0.96*	0.24 ± 1.12	*376.63*
***S. aureus*** **UAMS-1**	0.10 ± 1.35	*147.34*	0.01 ± 1.03	*21.30*
***P. aeruginosa*** **SAMMC-418**	0.00 ± 1.36	*0.98*	0.00 ± 1.60	*2.77*
***Leptin***				
**Differentiation control**	0.06 ± 1.02	*0.22*	0.90 ± 0.88	*93.90*
***S. aureus*** **UAMS-1**	0.01 ± 1.01	*0.05*	0.03 ± 0.60	*2.61*
***P. aeruginosa*** **SAMMC-418**	0.01 ± 1.00	*0.04*	0.00 ± 0.61	*0.23*

**PPAR**γ – peroxisome proliferator-activated receptor gamma.

Values represent the mean ± SD relative expression of genes as determined by the 2^-ΔCt^ method. Fold difference is the expression of normalized values as a ratio to undifferentiated growth controls.

**Table 2:** **Relative expression of genes involved in osteogenic differentiation**

	**Day 3**		**Day 7**	
	**Relative expression**	**Fold difference**	**Relative expression**	**Fold difference**
***ALP***				
**Differentiation control**	13.11 ± 0.34	*7.51*	7.69 ± 0.51	*14.55*
***S. aureus*** **UAMS-1**	3.23 ± 0.25	*1.85*	0.27 ± 0.38	*0.52*
***P. aeruginosa*** **SAMMC-418**	0.53 ± 0.35	*0.30*	0.07 ± 0.45	*0.13*
***RUNX2***				
**Differentiation control**	0.53 ± 0.82	*3.54*	0.91 ± 0.72	*2.59*
***S. aureus*** **UAMS-1**	0.21 ± 0.99	*1.42*	1.24 ± 0.70	*3.56*
***P. aeruginosa*** **SAMMC-418**	0.13 ± 0.62	*0.85*	1.35 ± 0.67	*3.86*
***OCN***				
**Differentiation control**	0.34 ± 0.36	*1.92*	31.91 ± 0.53	*10.42*
***S. aureus*** **UAMS-1**	0.01 ± 0.35	*0.08*	0.01 ± 0.86	*0.00*
***P. aeruginosa*** **SAMMC-418**	0.02 ± 0.27	*0.12*	0.00 ± 0.52	*0.00*

**ALP** – alkaline phosphatase; **RUNX2** – Runt-related transcription factor 2; **OCN** – bone gamma-carboxyglutamic acid-containing protein (osteocalcin).

Values represent the mean ± SD relative expression of genes as determined by the 2^-ΔCt^ method. Fold difference is the expression of normalized values as a ratio to undifferentiated growth controls.

**Figure 4:**
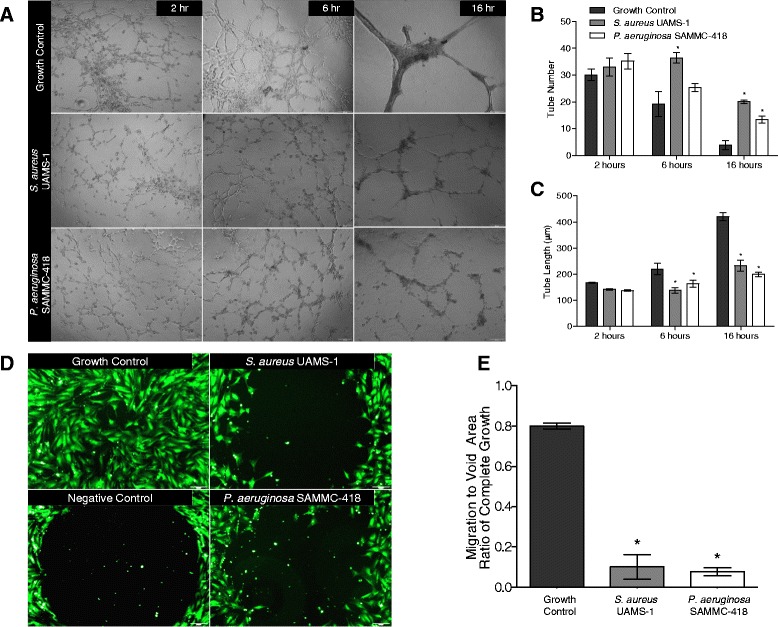
**Tube formation and migratory function of hBMSCs are differentially affected by the presence of soluble factors of biofilms. A)** Representative light microscopy images of hBMSCs after 2, 6 and 16 h of angiogenic differentiation on gel matrices (40x magnification) in the presence of BCM (25%) from *S. aureus* and *P. aeruginosa*. **B)** Quantification of capillary-like tube number and **C)** tube length (μm) between connectors using Image J software (n = 6 images per group).* *p* < 0.05 compared to growth control at each timepoint. **D)** Representative fluorescent microscopy images of hBMSCs stained with Vybrant® CFDA SE Cell Tracer dye (green, 40x magnification) after 3 days of migration into void area in the presence of BCM (25%) from *S. aureus* and *P. aeruginosa*. **E)** Quantification of cell migration into void area based on measurement of fluorescence and image thresholding, using Image J software; *p < 0.05 compared to growth control.

In congruence with their reduced adipogenic and osteogenic differentiation, the ability of hBMSCs to form capillary-like tubes was also inhibited in the presence of biofilm-derived factors (Figure 4A-C). In contrast to the untreated growth control group, which formed a stable tube network characterized by dense tubules forming as early as 6 hours on the gel matrices, hBMSCs exposed to BCM were unable to form mature tubule networks similar to that visualized for control groups within 16 hours (Figure 4A). Although, early exposures to BCM exhibited comparable tube numbers to control groups, as conventional stable networks formed with efficient merging in controls (no BCM exposure), networks with BCM exposure maintained a higher tube number, suggesting that the networks failed to mature at the expected rates (Figure 4B). Consistent with this, cells exposed to BCM had significantly decreased average tube lengths (Figure 4C), indicating that in the presence of BCM, the hBMSCs were delayed in their formation of established tube networks.

### Migration of hBMSCs is hindered by the presence of biofilm-derived factors

The ability of cells to migrate towards sites of injury is central to enacting their roles during the wound healing process. Using a void circle migration assay, the effect of BCM on the migratory capacity of hBMSCs was assessed. After 3 days, in the absence of BCM, cells were able to almost completely fill the void space in the standard migration assay (i.e. >80% confluence). In contrast, in the presence of BCM from *S. aureus* and *P. aeruginosa*, the migration of hBMSCs was significantly impaired (Figure 4D), with few to little cells migrating into the void space (i.e. <10%). Quantification of the ratio of migration, as determined by measuring the cell amount within the void area, demonstrated that on average, the exposure to BCM reduced the migration capacity by > 6 fold compared to the untreated growth control (Figure 4E).

### hBMSCs enhance the production of inflammatory and wound healing cytokines in response to soluble factors from biofilms

**Figure 5:**
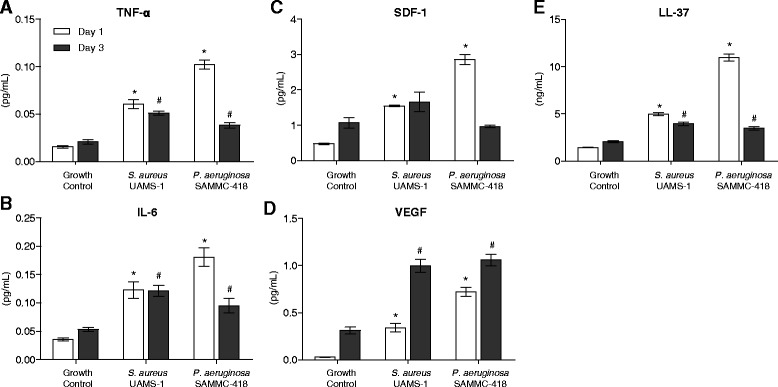
**Soluble factors from biofilms increase release of wound healing cytokines from hBMSCs.** Quantification of inflammatory cytokines TNF-α **(A)** and IL-6 **(B)**, wound healing cytokines SDF-1 **(C)** and VEGF **(D)**, and antimicrobial peptide LL-37 **(E)** in cell supernatants (media) by ELISA analysis following hBMSC exposure to BCM (25%) for 1 and 3 days. Protein levels were normalized, expressed as a ratio of specific cytokine concentration to total protein content (μg/mL, quantified via a bicinchoninic acid assay); *,# *p* < 0.05 compared to day 1 and 3 growth control, respectively.

MSCs modulate the wound environment through the release and activity of various cytokines. To evaluate the impact of BCM on the paracrine activity of hBMSCs, the levels of cytokines were quantified, including inflammatory cytokines (TNF-α, IL-6), wound healing cytokines (SDF-1, VEGF) and an antimicrobial peptide (LL-37) in hBMSC supernatants following exposure to BCM. In the presence of biofilm factors derived from *S. aureus* and *P. aeruginosa*, TNF-α and IL-6 were significantly increased at days 1 and 3 post-exposure compared to untreated control groups (Figure 5A-B, Additional file 3: Figure S3). Similarly, significant increases in the production of wound healing cytokines, SDF-1, VEGF, and antimicrobial peptide LL-37 by hBMSCs exposed to BCM were also observed (Figure 5C-E, Additional file 3: Figure S3).

## Discussion

In response to injury, the recruitment of host MSCs and their subsequent activity within wounds is a central part in the healing process. MSCs participate in wound healing directly, through differentiation and replacement of damaged tissues, and indirectly through paracrine activities, which can orchestrate a regenerative microenvironment [30-34], and modulate host immune responses [9,10,35]. However, within infected wounds, the persistence of microbial pathogens, in addition to the damaging environment caused by pathogen exposure, could differentially affect the properties of MSCs. Indeed, recent studies have demonstrated that the differentiation capacity of MSCs can be modulated in an inflammatory environment [36] as well as in the presence of purified cell wall components of bacterial pathogens [18,20,21]. Although these studies have provided insight into the interactions between MSCs and signals that would be present in an infected wound environment, evaluation of the effect of soluble factors of biofilms, the predominate mode of growth within tissues, on MSCs has not been previously reported.

Bacterial biofilms have been shown to directly disrupt the normal wound healing process in vitro and in vivo [37-40]. This is, in part, thought to be mediated through the activity of soluble bacterial products released from the biofilm on host cells. Recent studies comparing the effects of biofilm to planktonic cultures have shown that biofilms can induce distinctive inflammatory responses and differentially activate apoptosis in human cell lines [25,41]. Similarly, exposure of osteoblasts to soluble biofilm products resulted in increased apoptosis, decreased osteogenesis and enhanced release of factors promoting bone resorption [24]. Prior to the current study, the effect of biofilms on hBMSCs had not been studied. The present study showed early and pronounced negative effects on hBMSC viability along with increases in caspase activity following exposure to soluble factors from biofilms of *S. aureus* and *P. aeruginosa*. Comparisons of planktonic versus biofilm conditioned medias have demonstrated that there is a substantial overlap between the extracellular proteomes, with only a small minority of proteins unique to either phenotype [24,25,42]. Because biofilms are heterogeneous in their composition, having bacteria in the planktonic and biofilm state, the observed effects on hBMSCs are likely due to the cumulative effect of released proteins within conditioned medias expressed by both phenotypes, such as exoenzymes and exotoxins [24]. Future studies evaluating the soluble factors released by biofilms of these different bacterial species may provide further insight into the identity of these soluble factors as well as providing potential mechanisms for the loss of cell viability. Interestingly, exposure of hBMSCs to BCM of *P. aeruginosa* was observed to be much more toxic to cells compared to the conditioned media of *S. aureus*. Evaluation of viable bacteria within and released protein during biofilm growth revealed only moderate differences between the two strains, indicating that the observed differences on cell viability may not have been the result of higher protein concentrations or bacterial numbers within the biofilm, but due to differences in the factors expressed by the two bacterial species.

In addition to decreased cell viability, successful direct functional differentiation processes of MSCs were also significantly decreased in the presence of BCM. Specifically, the processes of adipogenesis (defined by transcriptional factors *PPAR*γ, *adiponectin,* and *leptin,* which are either regulators of adipocyte differentiation or secreted by mature adipocytes) and osteogenesis (defined by transcriptional factors *RUNX2, ALP* and *OCN,* which are essential for osteoblast differentiation, matrix elaboration, and bone mineralization and calcium ion homeostasis, respectively) were severely disrupted by the factors associated with both Gram-negative and Gram-positive bacterial biofilms. Further, functional assays supported this finding, showing little to no oil droplet formation or calcium deposition for cells undergoing adipogenesis and osteogenesis in the presence of BCM, respectively. Conversely, a previous study demonstrated that exposure of adipose-derived MSCs to heat-inactivated Gram-negative bacteria and LPS induced osteogenic differentiation but reduced adipogenic differentiation. Heat-inactivated Gram-positive bacteria showed similar results, while LTA had no impact on the cells [21]. In the current study, the loss in functional differentiation occurred with exposure to BCM of both strains, suggesting that the soluble products within the conditioned medias at the concentrations tested reduced the regeneration effects of MSCs. Although loss of cell viability may in part explain the reduced differentiation capacity of hBMSCs, use of multiple independent analysis requiring normalization of the data herein indicate a specific effect following treatment with BCM independent of cell loss.

Cellular migration and angiogenesis were assessed in the presence of BCM. Previous studies have observed increased cellular migration towards areas of *S. aureus*–infected cells [43] and increased cellular tube formation in the presence of bacteria [44], suggesting infectious pathogens promote migratory and angiogenic mechanisms of healing due to activation of the NF-κB pathway. Our results, however, indicate that migration, via a wound healing assay, and angiogenesis, via a tube formation assay, were significantly decreased with *S. aureus* and *P. aeruginosa* BCM present compared to growth controls. The explanation for this may possibly be due to the BCM pathogen-induced activation of specific toll-like receptors (TLRs). TLRs of MSCs have been identified as the recognition sites of bacterial structures, also known as pathogen-associated molecular patterns (PAMPs) [45,46]. For example, TLR4 is a receptor for LPS and TLR2 can bind bacterial lipoproteins. TLRs have been shown to affect proliferation and drive the critical stress responses of MSCs as well as disrupt normal differentiation pathways. In a recent study, MSCs that were treated with various TLR ligands showed induction of certain cytokines and chemokines, including TNF-α, chemokine ligand CCL2, CXCL10 interferon-gamma-inducible protein (IP10), IL-6, IL-8, interferon (IFN)1β, and NF-κB [17]. Futhermore, TLR stimulation also triggered enhanced migration of MSCs [47]. Activation of TLR3 in MSCs induced the release of trophic factors (IL-6, SDF-1, hepatocyte growth factor and VEGF), but inhibited proliferation and migration [48]. Identifying these distinct recognition pathways have led to the theory that MSCs can be polarized by downstream TLR signaling to a series of specific phenotypes, including a pro-inflammatory pathway (TLR4 initiated), or an immunosuppressive pathway (TLR3 initiated) [36]. The likely presence of multiple factors, representative of the planktonic and biofilm phenotypes, within the BCM suggests the possibility of TLR activation in MSCs, although the decrease in viability/proliferation with BCM may be adding a confounding factor that virtually eliminates healing functions. Future studies evaluating the role of TLRs in these processes are required to determine the exact mechanisms of action.

Despite functional assessments being hindered, trophic factors of hBMSCs in the presence of BCM were analyzed to determine if the innate healing cascade was activated during initial BCM exposure. MSC phenotype fate can be determined with minimal exposure to pathogens/agonists, with studies suggesting that as little as a one hour exposure can initiate changes, which has been used to mimic the gradient of danger signals that endogenous MSCs encounter and respond to at a distance from the injury site [36]. The present study provided a continuous exposure to soluble biofilm products to more closely mimic the clinical setting of pathogens in a wound. Initial exposure was assessed for the early onset of cellular death or phenotypic change, based on release of inflammatory cytokines and upregulation of wound healing and antimicrobial cytokines. TNF-α and IL-6, both cytokines suggestive of a distressed, pro-inflammatory environment, were upregulated in the presence of BCM. TNF-α is a key regulator of the NF-κB pathway, which, as mentioned previously, regulates genes that influence migration, proliferation, differentiation and inflammation. Interestingly, TNF-α has been shown to stimulate MSC proliferation through the upregulation of cyclin D [49]; however, a severe decrease in proliferation and viability was observed after exposure to BCM even though TNF-α was significantly increased. IL-6 expression in cells, which typically exhibits immunomodulatory [45,50] and anti-inflammatory [51] responses, was similarly upregulated in hBMSCs in the presence of BCM. Previous reports demonstrate SDF-1 upregulation during inflammation processes in MSCs [11,12,52]. Our results illustrate that in the presence of soluble factors from biofilms, there was a significant increase in secreted SDF-1 protein. Secretion of VEGF, an important factor for mediation of angiogenesis, is increased in MSCs (specifically, adipose-derived stem cells) which were exposed to wound exudates [13]. This process is part of a healing cascade during which MSCs exhibit angiogenic potential during inflammatory processes to contribute to oxygenation of a wound [53]. The exposure of hBMSCs to BCM provided a similar result, with increased secreted VEGF. Human cathlicidin antimicrobial peptide hCAP-18/LL-37 has been identified as one of the factors that defines the antimicrobial activity of MSCs [54]. The exposure of hBMSCs to BCM significantly increased the levels of secreted LL-37, suggesting that initial short-term presence of soluble factors from biofilms allowed for anticipated increases in antimicrobial activity. The assessment of these mediators indicates that the healing signals of hBMSCs followed the expected inflammatory responses according to the paracrine activity assessed.

## Conclusions

Collectively this study demonstrates that in the presence of soluble factors released by biofilms, the regenerative capacity of MSCs is hindered or lost, while maintaining a secretory phenotype. The main findings of this study are that soluble factors released by mature biofilms of clinically relevant pathogens can induce initial inherent healing pathways; however, there is a negative impact on the viability of MSCs partially by induction of apoptosis, a hindrance of healing mechanisms of migration and angiogenesis, and incomplete multi-differentiation potential. The combination of these responses to biofilms gives insight to the non-healing aspect of chronic wounds, which typically have a strong biofilm presence; furthermore, these facts indicate that the use of cell-based therapies for the treatment of chronic wounds may have a less than desired effect for tissue regeneration in the context of infection. Future work may include the elucidation of the mechanisms of action of biofilms on MSCs to develop optimized therapies allowing MSCs to function in the presence of infection.

## Additional files

Additional file 1: Figure S1. Concentration dependent effect of biofilm factors on hBMSC viability. **(A)** Cell viability (performed via a LIVE/DEAD© assay kit) of hBMSCs following 24-hr exposure to supplemented DMEM containing increasing concentrations (0-100%) PBS (vehicle control), *S. aureus* UAMS-1 biofilm-conditioned media (BCM), or *P. aeruginosa* SAMMC- 418 biofilm-conditioned media (BCM). Dashed line represents viability of untreated growth control. **(B)** Representative fluorescent images of viable hBMSCs following exposure to increasing concentrations of BCM after 24 hours, stained with calcein (green, 40x magnification). Losses in viability became noticeably evident at 50% (after 24-hr exposure) and therefore, 25% BCM was used in all future studies to assess hBMSC function without a substantial loss of viability. Additional file 2: Figure S2. Quantification of adipogenic and osteogenic differentiation proteins of hBMSCs following exposure to soluble biofilm factors. Protein was isolated and quantified from pooled (n = 3) lysates of cultured hBMSCs that were exposed to soluble biofilm factors from *S. aureus* UAMS-1 and *P. aeruginosa* SAMMC-418 under adipogenic **(A)** and osteogenic **(B)** conditions. Cells were exposed for 7 days. Under adipogenesis, adiponectin, PPARγ and leptin proteins were analyzed and expression was normalized to GAPDH. Under osteogenesis, RUNX2, osteocalcin and ALP were analyzed and expression was normalized to GAPDH. All differentiation proteins were lower in samples that were exposed to biofilm factors in comparison to differentiation controls, regardless of whether cells were undergoing adipogenesis or osteogenesis. Additional file 3: Figure S3. Cytokine concentrations released from hBMSCs following biofilm factor exposure. Cytokine levels were calculated based on ELISA analysis of an aliquot of media (total volume 0.5mL supernatant) taken from cells exposed to BCM. Values obtained were correlated to a standard curve of known concentrations provided by the manufacturers for each respective cytokine. Absolute values from cell monolayers and normalized values (to total protein content) are presented. Fold increases above normalized growth control values at each timepoint were calculated.
