# Supplementary material for: Soluble factors from biofilms of wound pathogens modulate human bone marrow-derived stromal cell differentiation, migration, angiogenesis, and cytokine secretion
Source: BMC Microbiol. 2015 Mar 28;15:75. doi: 10.1186/s12866-015-0412-x (PMC4381664; doi:10.1186/s12866-015-0412-x)
Supplement: Additional file 1: Figure S1. — Concentration dependent effect of biofilm factors on hBMSC viability. (A) Cell viability (performed via a LIVE/DEAD© assay kit) of hBMSCs following 24-hr exposure to supplemented DMEM containing increasing concentrations (0-100%) PBS (vehicle control), S. aureus UAMS-1 biofilm-conditioned media (BCM), or P. aeruginosa SAMMC- 418 biofilm-conditioned media (BCM). Dashed line represents viability of untreated growth control. (B) Representative fluorescent images of viable hBMSCs following exposure to increasing concentrations of BCM after 24 hours, stained with calcein (green, 40x magnification). Losses in viability became noticeably evident at 50% (after 24-hr exposure) and therefore, 25% BCM was used in all future studies to assess hBMSC function without a substantial loss of viability. [file 12866_2015_412_MOESM1_ESM.pdf]

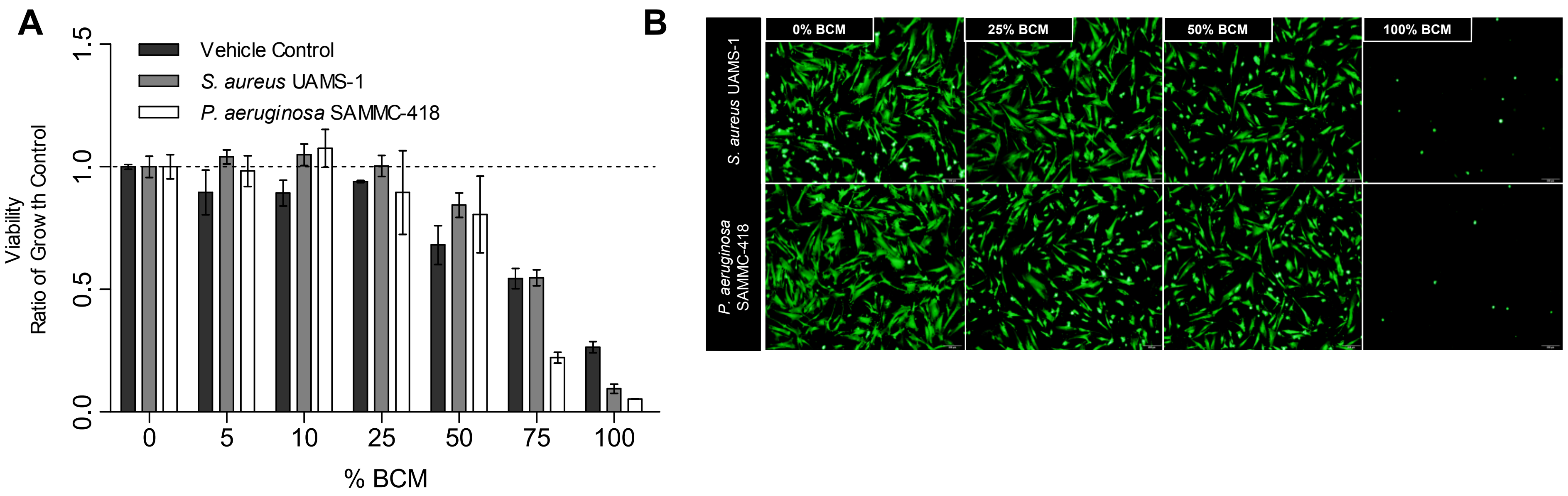

Supplemental Figure 1. Concentration dependent effect of biofilm factors on hBMSC viability. **(A)** Cell viability (performed via a LIVE/DEAD© assay kit) of hBMSCs following 24-hr exposure to supplemented DMEM containing increasing concentrations (0-100%) PBS (vehicle control), *S. aureus* UAMS-1 biofilm-conditioned media (BCM), or *P. aeruginosa* SAMMC-418 biofilm-conditioned media (BCM). Dashed line represents viability of untreated growth control. **(B)** Representative fluorescent images of viable hBMSCs following exposure to increasing concentrations of BCM after 24 hours, stained with calcein (green, 40x magnification). Losses in viability became noticeably evident at 50% (after 24-hr exposure) and therefore, 25% BCM was used in all future studies to assess hBMSC function without a substantial loss of viability.
