# Supplementary material for: Soluble factors from biofilms of wound pathogens modulate human bone marrow-derived stromal cell differentiation, migration, angiogenesis, and cytokine secretion
Source: BMC Microbiol. 2015 Mar 28;15:75. doi: 10.1186/s12866-015-0412-x (PMC4381664; doi:10.1186/s12866-015-0412-x)
Supplement: Additional file 2: Figure S2. — Quantification of adipogenic and osteogenic differentiation proteins of hBMSCs following exposure to soluble biofilm factors. Protein was isolated and quantified from pooled (n = 3) lysates of cultured hBMSCs that were exposed to soluble biofilm factors from S. aureus UAMS-1 and P. aeruginosa SAMMC-418 under adipogenic (A) and osteogenic (B) conditions. Cells were exposed for 7 days. Under adipogenesis, adiponectin, PPARγ and leptin proteins were analyzed and expression was normalized to GAPDH. Under osteogenesis, RUNX2, osteocalcin and ALP were analyzed and expression was normalized to GAPDH. All differentiation proteins were lower in samples that were exposed to biofilm factors in comparison to differentiation controls, regardless of whether cells were undergoing adipogenesis or osteogenesis. [file 12866_2015_412_MOESM2_ESM.pdf]

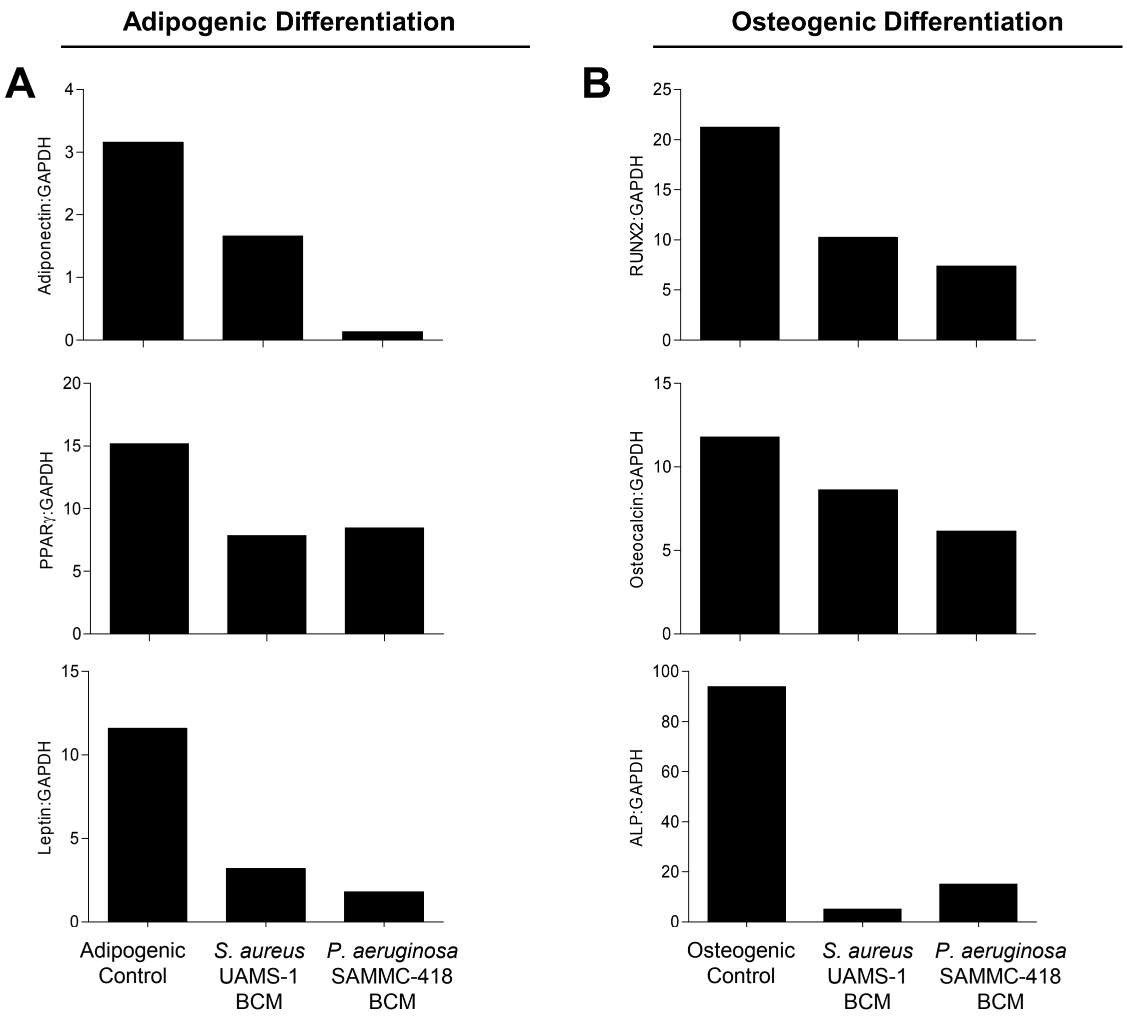

Supplemental Figure 2. Quantification of adipogenic and osteogenic differentiation proteins of hBMSCs following exposure to soluble biofilm factors. Protein was isolated and quantified from pooled (n=3) lysates of cultured hBMSCs that were exposed to soluble biofilm factors from *S. aureus* UAMS-1 and *P. aeruginosa* SAMMC-418 under adipogenic **(A)** and osteogenic **(B)** conditions. Cells were exposed for 7 days. Under adipogenesis, adiponectin, PPARγ and leptin proteins were analyzed and expression was normalized to GAPDH. Under osteogenesis, RUNX2, osteocalcin and ALP were analyzed and expression was normalized to GAPDH. All differentiation proteins were lower in samples that were exposed to biofilm factors in comparison to differentiation controls, regardless of whether cells were undergoing adipogenesis or osteogenesis.
