# Supplementary material for: Soluble factors from biofilms of wound pathogens modulate human bone marrow-derived stromal cell differentiation, migration, angiogenesis, and cytokine secretion
Source: BMC Microbiol. 2015 Mar 28;15:75. doi: 10.1186/s12866-015-0412-x (PMC4381664; doi:10.1186/s12866-015-0412-x)
Supplement: Additional file 3: Figure S3. — Cytokine concentrations released from hBMSCs following biofilm factor exposure. Cytokine levels were calculated based on ELISA analysis of an aliquot of media (total volume 0.5mL supernatant) taken from cells exposed to BCM. Values obtained were correlated to a standard curve of known concentrations provided by the manufacturers for each respective cytokine. Absolute values from cell monolayers and normalized values (to total protein content) are presented. Fold increases above normalized growth control values at each timepoint were calculated. [file 12866_2015_412_MOESM3_ESM.pdf]

	Day 1								
**Cytokine**	**Growth Control**			***S. aureus* UAMS-1**			***P. aeruginosa* SAMMC-418**		
	Absolute	Normalized Value (/mL)	Fold Increase of Normalized Value	Absolute	Normalized Value (/mL)	Fold Increase of Normalized Value	Absolute	Normalized Value (/mL)	Fold Increase of Normalized Value
TNF-α (pg)	15.07	0.016	*1*	15.64	0.060	*3.75*	12.08	0.102	*6.38*
IL-6 (pg)	34.52	0.036	*1*	31.73	0.123	*3.42*	21.38	0.181	*5.03*
SDF-1 (pg)	462.5	0.477	*1*	398.67	1.542	*3.23*	337.58	2.858	*5.99*
VEGF (pg)	27.98	0.029	*1*	88.22	0.341	*11.75*	85.19	0.721	*24.86*
LL-37 (ng)	1.40	1.448	*1*	1.28	4.962	*3.42*	1.29	10.959	*7.57*

	Day 3								
**Cytokine**	**Growth Control**			***S. aureus* UAMS-1**			***P. aeruginosa* SAMMC-418**		
	Absolute	Normalized Value (/mL)	Fold Increase of Normalized Value	Absolute	Normalized Value (/mL)	Fold Increase of Normalized Value	Absolute	Normalized Value (/mL)	Fold Increase of Normalized Value
TNF-α (pg)	13.41	0.021	*1*	16.76	0.051	*2.43*	14.25	0.038	*1.81*
IL-6 (pg)	34.27	0.053	*1*	39.89	0.121	*2.28*	35.39	0.095	*1.79*
SDF-1 (pg)	693.92	1.071	*1*	547.75	1.663	*1.55*	360	0.964	*0.90*
VEGF (pg)	203.87	0.315	*1*	329.10	0.999	*3.17*	395.06	1.058	*3.36*
LL-37 (ng)	1.33	2.052	*1*	1.30	3.945	*1.92*	1.30	3.484	*1.69*

Supplemental Figure 3. Cytokine concentrations released from hBMSCs following biofilm factor exposure. Cytokine levels were calculated based on ELISA analysis of an aliquot of media (total volume 0.5mL supernatant) taken from cells exposed to BCM. Values obtained were correlated to a standard curve of known concentrations provided by the manufacturers for each respective cytokine. Absolute values from cell monolayers and normalized values (to total protein content) are presented. Fold increases above normalized growth control values at each timepoint were calculated.
